# Phosphorylation in intrinsically disordered regions regulates the activity of Neurogenin2

**DOI:** 10.1186/s12858-014-0024-3

**Published:** 2014-11-06

**Authors:** Gary S McDowell, Christopher J Hindley, Guy Lippens, Isabelle Landrieu, Anna Philpott

**Affiliations:** 1 Department of Oncology, MRC/Hutchison Research Centre, University of Cambridge, Cambridge Biomedical Campus, Cambridge CB2 0XZ, UK; 2 CNRS, Université de Lille 1, Villeneuve d¿Ascq, UMR 8576, France; 3 Current address: Center for Regenerative and Developmental Biology, Department of Biology, Tufts University, 200 Boston Avenue, Medford 02155, MA, USA; 4 Current address: The Gurdon Institute, University of Cambridge, Tennis Court Road, Cambridge CB2 1QN, UK

**Keywords:** Phosphorylation, Intrinsic disorder, Protein NMR, Xenopus laevis, Neurogenin, Protein stability, Transcription factor, bHLH proteins, Protein folding

## Abstract

**Background:**

Neuronal differentiation is largely under the control of basic Helix-Loop-Helix (bHLH) proneural transcription factors that play key roles during development of the embryonic nervous system. In addition to well-characterised regulation of their expression, increasing evidence is emerging for additional post-translational regulation of proneural protein activity. Of particular interest is the bHLH proneural factor Neurogenin2 (Ngn2), which orchestrates progression from neural progenitor to differentiated neuron in several regions of the central nervous system. Previous studies have demonstrated a key role for cell cycle-dependent multi-site phosphorylation of Ngn2 protein at Serine-Proline (SP) sites for regulation of its neuronal differentiation activity, although the potential structural and functional consequences of phosphorylation at different regions of the protein are unclear.

**Results:**

Here we characterise the role of phosphorylation of specific regions of Ngn2 on the stability of Ngn2 protein and on its neuronal differentiation activity *in vivo* in the developing embryo, demonstrating clearly that the location of SP sites is less important than the number of SP sites available for control of Ngn2 activity *in vivo*. We also provide structural evidence that Ngn2 contains large, intrinsically disordered regions that undergo phosphorylation by cyclin-dependent kinases (cdks).

**Conclusions:**

Phosphorylation of Ngn2 occurs in both the N- and C-terminal regions, either side of the conserved basic Helix-Loop-Helix domain. While these phosphorylation events do not change the intrinsic stability of Ngn2, phosphorylation on multiple sites acts to limit its ability to drive neuronal differentiation *in vivo*. Phosphorylated regions of Ngn2 are predicted to be intrinsically disordered and cdk-dependent phosphorylation of these intrinsically disordered regions contributes to Ngn2 regulation.

## Background

During normal embryonic development, the tight regulation of cell fate decisions is absolutely required and differentiated phenotype is generally conferred by the transcription factor profile. In some cases a complete change of cellular identity can be driven by ectopic expression of one or a combination of such factors. For instance, fibroblasts can be reverted to pluripotency by introduction of the transcription factors Oct4, Sox2, Klf4 and c-Myc [1]. Studies have also highlighted the potential of the basic Helix-Loop-Helix (bHLH) family of transcription factors to induce fate reprogramming of mature cells, converting cells from one differentiated phenotype into another: for example, exogenous MyoD is capable of reprogramming mouse fibroblasts to myocytes [2], and Ngn3, Pdx1 and MafA can convert liver cells into pancreatic beta cells *in vivo*[3]. While transcription factor expression and activity can be manipulated *in vitro* and *in vivo* to generate cells of a specific phenotype, it is clear that *in vivo* such transcription factor networks are tightly regulated to maintain phenotypic stability, and prevent inappropriate activation that might lead to dysregulation of the maintenance of cell fate. Whilst most efforts have focused on elucidating regulation of lineage-specific transcription factors at the level of gene expression, emerging evidence increasingly points to post-translational modification as a key regulator of transcriptional networks in response to the cellular environment [4].

Neurogenin2 (Ngn2) is a bHLH transcription factor that regulates the transition from neural progenitor to differentiating neuron. For instance, Ngn2 is involved in the formation of sensory neurons from the epibranchial placode [5] and neurogenesis in dorsal root ganglia [6], where it acts to determine the fate of progenitor cells as neuronal precursors and also to repress glial cell fates using separate mechanisms [7],[8]. Furthermore, cell cycle exit and cell motility are regulated by Ngn2 [9], while experimentally, differentiation of neural stem cells can be induced by transfection of Ngn2 [10],[11]. To undertake these roles, Ngn2 acts to upregulate a large number of downstream targets, including a cascade of additional bHLH transcription factors, such as NeuroD, as well as structural and functional genes associated with mature neuronal activity [12]. For transcriptional activity at its targets, Ngn2 has an obligate heterodimeric DNA binding partner, E12/E47, and this heterodimer binds to DNA, recruiting transcriptional coactivators such as p300/CBP and Brg1 to facilitate target upregulation [13],[14]. However, Ngn2 is expressed in neural progenitor cells prior to their differentiation [5], implying that its neurogenic activity must be suppressed until a permissive environment exists. Such regulation is likely to occur at the level of post-translational control of protein activity, which in turn is likely to be both dependent on and a regulator of protein tertiary and quaternary structure, although the higher order structure of Ngn2 is poorly characterised.

Proteins carry out various roles in the cell as a function of their structure. Enzymes require specific active site conformations in order to carry out catalysis and structural proteins require particular conformations to achieve mechanical strength. However it has become apparent that many functional proteins are, in fact, not natively folded at all times in the cell [15]. Proteins that are natively unfolded and lacking regular structure are termed intrinsically disordered (ID) proteins [16]. ID proteins show a lack of hydrophobicity [17] and extreme isoelectric point (pI) values due to a large net charge from a high prevalence of charged residues (see Table one in Weinreb et al., [18]). It has also been suggested that there is an increased rate of evolution in ID segments and proteins [19]. Therefore it may be that in these regions there is a lack of function allowing unconstrained mutation; or perhaps the function of the protein requires disorder itself and so a lack of evolutionary constraint on specific sequence allows a larger amount of variability. An investigation of the gene ontology terms associated with ID proteins highlights a significant number of proteins that are involved in DNA-binding, transcription activation and that act as transcription factors [20]. In these cases, intrinsic disorder would appear to regulate protein-protein and protein-nucleic acid binding interactions, as thermodynamically this gives flexibility to signaling processes and the assembly of complexes in the cell. The free energy of binding is counteracted by the free energy required to fold the structure and so overall there is only a small free energy change [21].

While such a small free energy change would be predicted to allow only a low affinity of binding, there is a trade-off achieved with high specificity, through a requirement for the correct binding interactions and easy reversibility [22]. So small are the energy differences involved, that this may result in minor changes to the structure of a protein, but dramatic alterations to the formation of multi-factor complexes. For example, this may allow fine tweaking of transcription dynamics, to form promoter complexes with DNA-binding cycles of various time periods leading to differential activation of various downstream targets [23],[24]. ID proteins exhibit less transcriptional noise in expression than structured proteins and so appear to be tightly regulated at the point of their destruction [25], which also may further lead to tight control of downstream target gene expression. There is a high correlation between specific amino acid composition of ID proteins and phosphorylation sites, suggesting that phosphorylation may be promoted in disordered regions [26]. Furthermore, ID proteins are targeted by twice as many kinases as structured proteins [25]. Many of those kinases whose substrates are mainly unstructured proteins also tend to be regulated in a cell cycle-dependent manner [25].

Ngn2 exhibits some of the features common to ID proteins: there are a large number of charged residues leading to a low level of hydrophobicity [27] and the protein runs at a higher molecular mass (around 36 kDa) than its predicted molecular mass (23.4 kDa) [18]. Ngn2 also shares many similarities with ID proteins with regard to amino acid composition [27], function [20] and stabilization upon the binding of other cofactors [28],[29]. bHLH proteins show significant sequence homology in the bHLH domain only [30] and large sequence variability in the flanking regions, as is found in the evolutionary comparison of related ID proteins [19], again indicating the potential for large disordered regions in Ngn2. There is no published structural information for Ngn2 but a limited number of crystal structures are available for bHLH family homologues such as MyoD and NeuroD, bound to E47 as a heterodimer to DNA [31],[32]. bHLH protein crystal structures exclude the N- and C-terminal regions outside of the bHLH domain [31], as these regions are predicted to be disordered and thus will reduce the ability to purify, and therefore crystalise, the protein [33].

The stability of many bHLH proteins is regulated by phosphorylation events, e.g. MyoD [34]. Our previous work has highlighted that Ngn2 is a highly unstable protein [28],[35]-[37], which appears to be highly phosphorylated in a cell cycle-dependent manner at serine-proline (SP) sites [38],[39]. The relationship between the stability of Ngn2 and its phosphorylation status has been only briefly explored previously [28],[38] but a direct link between phosphorylation at SP sites and the stability of Ngn2 has not been investigated. In this work we explore the effects of phosphorylation at specific SP sites of Ngn2 on the activity and structure of the protein. We find that phosphorylation of Ngn2 has no significant effect on its intrinsic stability. Mutation of phosphorylation sites in both the N- and C-terminal regions contributes to enhanced Ngn2 neuronal differentiation activity and further mutational analysis confirms that it is the number, and not position, of phosphorylation sites available that controls protein activity *in vivo.* Finally, we also present the first structural validation by NMR of full-length Ngn2 protein, and directly demonstrate phosphorylation in the N-terminal region of mNgn2 by NMR spectroscopy.

## Results

### Mutation of conserved and non-conserved SP phosphorylation sites does not significantly alter Ngnprotein half-life in vitro

If specific phosphorylation sites act to regulate Ngn2 activity then we would expect such sites to be highly conserved across species, in line with the conservation of Ngn2 function. All available NCBI Ngn2 protein sequences were aligned using the Clustal W2 multiple sequence alignment tool (Additional file 1: Figure S1), and compared to *Xenopus laevis* Ngn2 (xNgn2), where multi-site phosphorylation and stability have been best characterised [28],[35]-[39]. The Ngn2 bHLH domain was defined by homology to the MyoD and NeuroD bHLH domains as lying between glycine 72 and leucine 139 of the xNgn2 sequence ([30], Additional file 1: Figure S1, green box). Compared to human Ngn2 (hNgn2), the sequence most divergent from xNgn2, the xNgn2 bHLH domain shows 84% conservation and 99% similarity and thus appears to be highly conserved, in agreement with previously published studies (reviewed in [30]). By contrast, the N-terminal region of xNgn2 shows 24% conservation and 70% similarity and the C-terminal region of xNgn2 shows 31% conservation and 74% similarity to hNgn2. The N- and C-terminal regions are therefore not as highly conserved as the bHLH domain but both show a similar extent of conservation compared to each other.

Ngn2 has previously been shown to be phosphorylated on serines of serine-proline (SP) sites [38],[39], while threonine-proline (TP) sites may also be potential sites of phosphorylation. There are no SP or TP sites present in the bHLH domain of any species. Surprisingly, given the poor conservation of sequence overall, three of the four SP sites (serines 172, 181 and 184) in the C-terminal region of xNgn2 are highly conserved with all other species whilst the other SP site is close in the primary sequence to a site conserved in other species. These sites include the two residues identified as glycogen synthase kinase 3? (GSK3?) phosphorylation sites in mNgn2 [40]. By contrast, despite the presence of 5 SP sites in the N-terminal region of xNgn2, a highly conserved SP site is seen in the N-terminal region of other species but this is not conserved in xNgn2. Although we have previously shown that in general it is the number of SP site phosphorylation events that is critical for regulating Ngn2 function *in vitro* rather than their precise location [38],[39], nevertheless, the conservation of SP site locations raises the possibility that SP or TP phosphorylation sites in the C-terminal region may be particularly important for the regulation of Ngn2 function. We have explored this possibility here by investigating further the protein stability and function of xNgn2 mutants where specific combinations of SP sites are mutated to prevent their phosphorylation.

To assess the role of SP site phosphorylation in the regulation of Ngn2 stability and function, we generated mutant versions of xNgn2 in which either N- or C-terminal region SP sites were mutated to Alanine-Proline (AP) to generate NT-S-AxNgn2 and CT-S-AxNgn2, respectively (see Additional file 2: Figure S2 for a summary of SP site mutants used). We first analysed protein stability in mitotic *Xenopus* egg extracts that have been previously shown to maximally phosphorylate Ngn2 at SP sites [38]. NT-S-AxNgn2 and CT-S-AxNgn2 protein stabilities were compared to those of wild-type xNgn2 and a mutant version of xNgn2 in which all SP sites are mutated to AP and which demonstrates a dramatically increased neuronal differentiation activity *in vivo* in *Xenopus* frog embryos (9S-AxNgn2, see Additional file 2: Figure S2 and [38]).

**Figure 1:**
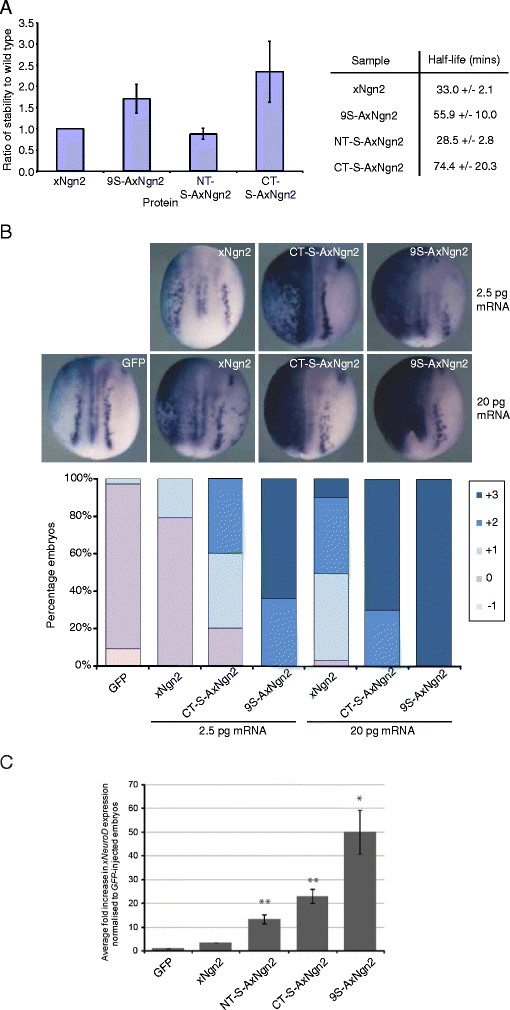
**Phosphorylation at SP sites in both the N- and C-terminal regions regulates xNgn2 activity. (A)**^35^S-labelled IVT xNgn2, or the indicated Serine-Proline to Alanine-Proline xNgn2 mutants, full phosphomutant (9S-AxNgn2), N-terminal region (NT-S-AxNgn2) or C-terminal region (CT-S-AxNgn2), were added to *Xenopus laevis* mitotic egg extracts and incubated at 21°C. Samples were taken at 0, 15, 30, 45, 60, 75, 90 and 120 mins and separated on 15% SDS-PAGE gels. Gels were analyzed by quantitative phosphorimaging analysis, calculating the average stabilization relative to wild-type xNgn2 within mitotic extract. Half-lives were calculated using first-order rate kinetics, and errors calculated using the Standard Error of the Mean (SEM). **(B)** Embryos were injected into 1 cell of 2 cells with the indicated amount of mRNA encoding GFP, xNgn2, CT-S-AxNgn2 or 9S-AxNgn2, injected side to the left. Embryos were fixed at stage 15 and subjected to *in situ* hybridization for neural ß-tubulin expression before being scored for increased neurogenesis on the injected side compared to the uninjected side on a scale of 0¿3 [38]. The experiment was performed in duplicate (n?=?17-37). **(C)** 1 cell-stage embryos were injected with 20 pg of mRNA encoding GFP, xNgn2, NT-S-AxNgn2, CT-S-AxNgn2 or 9S-AxNgn2, harvested at stage 15 and expression of xNeuroD analysed by qPCR (5 embryos per sample, n?=?3).

To investigate the effect of mutation of N- and C-terminal region SP sites on protein stability, ^35^S-radiolabelled *in vitro* translated (IVT) xNgn2, 9S-AxNgn2, NT-S-AxNgn2 and CT-S-AxNgn2 were added to *Xenopus* mitotic egg extract and samples removed at increasing timepoints. Proteins were separated by 15% SDS-PAGE and subjected to autoradiography to allow measurement of protein levels over time and calculation of the half-life for degradation using first-order rate kinetics (Figure 1A) [28],[35]. In this assay, xNgn2 had a half-life of 33.0 +/? 2.1 mins. Although the trend is towards increased stability, 9S-AxNgn2 shows no statistically significant difference compared to wild-type Ngn2, with a half-life of 55.9 +/? 10.0 mins. NT-S-AxNgn2 has a half-life of 28.5 +/? 2.8 mins, while CT-S-AxNgn2 has a half-life of 74.4 +/? 20.3 mins. Both mutant versions therefore tend towards increased stability compared to wild-type xNgn2, but again variability in repeat assays means that no statistically significant difference in stability was observed. Therefore, we have directly demonstrated that phosphorylation status of Ngn2 does not directly control its stability in *Xenopus*.

### SP sites in both the N- and C-terminal regions regulate the activity of xNgn2

Previous work has indicated that preventing xNgn2 phosphorylation on all 9 SP sites enhances its ability to drive neuronal differentiation *in vitro* and *in vivo*[38],[39]. We have observed phosphorylation of xNgn2 at SP sites in both the N- and C-terminal regions following incubation in *Xenopus* mitotic egg extract [38]. Assaying the activity of a sequential mutant series of mouse Ngn2 (mNgn2) where SP sites were additively mutated from the C-terminus (mutating the first C-terminal SP in mutant 1, the first and second in mutant 2, the first, second and third in mutant 3 etc.) showed an incremental increase in neuronal differentiation activity *in vitro* with the loss of each additional SP site [38],[39]. In addition, analysis of the *in vivo* neuronal differentiation activity of a single SP site knock-in series allowed us to determine that phosphorylation at individual SP sites could not account for regulation of the neuronal differentiation activity of xNgn2 [38]. However, these analyses did not allow us to determine the contribution of the N- and C-terminal region SP sites to the phospho-regulation of the neuronal differentiation activity of mNgn2, nor whether one region played a more prominent regulatory role than the other. mNgn2 has only two N-terminal SP sites and previous assays [38] showed in fact that their mutation had a minimal effect on the differentiation of P19 cells, which respond to overexpression of proneural proteins by undergoing neuronal differentiation [41]. This points to increased prominence for C-terminal phosphorylation events for Ngn2 regulation. However, it was unclear whether the mNgn2 N-terminal SP sites had only minimal effects on the neuronal dif-ferentiation-inducing ability of Ngn2 because there are only two of them or because phosphorylation of sites in the N-terminus of Ngn2 plays an intrinsically lesser role in regulating Ngn2 activity than phosphorylation of sites in the C-terminus for domain structure/function reasons. By contrast to mNgn2, xNgn2 has five SP sites in the N-terminus. We investigated whether the larger number of potential phosphorylation sites in xNgn2 compared to mNgn2 results in a greater contribution of N-terminal phosphorylation to regulation of xNgn2 activity relative to mNgn2, or whether N-terminal phosphorylation, regardless of number of sites available, plays little role in phospho-regulation of the neuronal differentiation activity of Ngn2 protein.

To assess whether preventing phosphorylation at the conserved SP sites in the C-terminus of xNgn2 alone was sufficient to maximally activate the protein, we compared the neuronal differentiation activity of xNgn2, CT-S-AxNgn2 and 9S-AxNgn2 *in vivo* in *Xenopus* embryos. To this end, mRNAs encoding xNgn2, CT-S-AxNgn2 and 9S-AxNgn2 were generated and overexpressed in 1 cell of 2 cell *Xenopus* embryos, and an assessment of ectopic neurogenesis was performed as previously described, comparing the injected and the uninjected side [38]. As expected, 9S-AxNgn2 had significantly higher neuronal differentiation activity than wild-type xNgn2 [38],[39]. The overexpression of CT-S-AxNgn2 resulted in a level of ectopic neurogenesis intermediate between that of overexpressed wild-type xNgn2 and 9S-AxNgn2 (Figure 1B), indicating that phosphorylation on the N-terminus of xNgn2 does indeed contribute to limiting xNgn2 neuronal differentiation activity. To further investigate the neuronal differentiation activity of the mutants *in vivo*, we then quantified the expression of the direct downstream xNgn2 target xNeuroD by qRT-PCR in response to overexpression of xNgn2, NT-S-AxNgn2, CT-S-AxNgn2 and 9S-AxNgn2 in 1 cell-stage *Xenopus* embryos (Figure 1C). Both NT-S-AxNgn2 and CT-S-AxNgn2 showed an intermediate neuronal differentiation activity between that of xNgn2 and 9S-AxNgn2, demonstrating that phosphorylation at SP sites in both the N- and C-terminal regions of xNgn2 contributes to regulating its transcriptional activity. Therefore, in addition to our previous work highlighting the importance of the number of SP sites for regulation of neuronal differentiation activity, we show clearly here that SP sites in both the N- and C-terminal domains can contribute to regulation of protein function.

### In vivo activity of xNgnis a semi-quantitative measure of phospho-site availability

**Figure 2:**
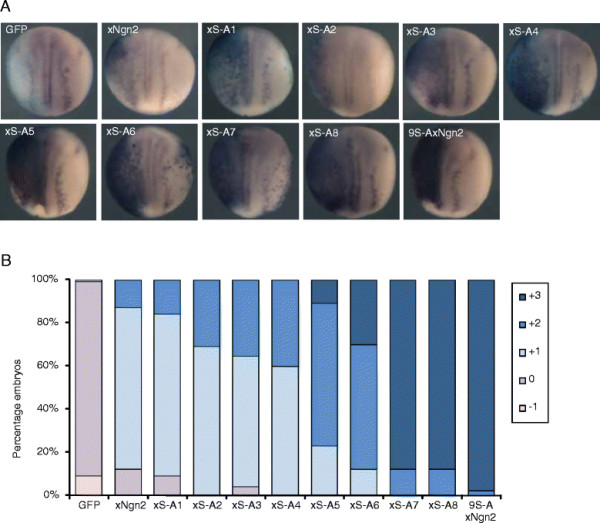
**SP site availability semi-quantitatively controls neuronal differentiation activity of xNgn2*in vivo*. (A)** Embryos were injected into 1 cell of 2 cells with 20 pg of mRNA encoding GFP, xNgn2, or the indicated mutant version of xNgn2 from the cumulative mutant series (see Additional file 2: Figure S2), injected side to the left. Embryos were fixed at stage 15 and subject to *in situ* hybridization for neural ß-tubulin expression. **(B)** Scoring of ectopic neurogenesis on the injected side of embryos from **(A)** on a scale of ?1 - +3 [38]. The experiment was performed in triplicate (n?=?47-87).

P19 embryonal carcinoma cells respond to ectopic Ngn2 expression by undergoing neuronal differentiation *in vitro*[41]. Our previous data in P19 cells with a cumulative phospho-mutant series of mNgn2 proteins (where SP sites are additively mutated from the C-terminus: mutant 1, most C-terminal SP site mutated, mutant 2, the two SP sites nearest the C-terminus are mutated, etc.) had suggested that the number of SP sites available for phosphorylation act semi-quantitatively to regulate the neuronal differentiation activity of Ngn2 [38],[39]. The results presented above support the hypothesis that phospho-regulation of Ngn2 is not strictly dependent on the position or level of conservation of the SP site that is modified but rather on the number of SP sites available. To confirm this *in vivo*, we overexpressed a cumulative phospho-mutant series of xNgn2 proteins, this time where SP sites were additively mutated from the N-terminus (mutant 1, most N-terminal SP site mutated, mutant 2, the two SP sites nearest the N-terminus are mutated, etc.; Additional file 2: Figure S2, as opposed to mutation from the C-terminus for mNgn2 as described above [38]), in 1 cell of 2 cell *Xenopus* embryos, to semi-quantitatively assay the multi-site phos-pho-regulation of xNgn2?s neuronal differentiation activity *in vivo*. Embryos were subjected to *in situ* hybridization for neural ?-tubulin expression and the injected and uninjected sides compared as above (Figure 2). As additional SP sites were mutated from the N-terminus, we saw that the neuronal differentiation activity of the mutant xNgn2 proteins progressively increased, finally reaching maximal activity when all 9 SP sites were mutated in 9S-AxNgn2. Strikingly, when taken with data demonstrating a gradual increase in mNgn2 activity in P19 cells *in vitro* as SP sites are cumulatively mutated from the C-terminus [38], these data demonstrate conclusively that the precise location of SP sites is not of major importance for the phospho-regulation of Ngn2 activity, but rather it is the number of SP sites available for modification that is critical for controlling Ngn2 activity *in vivo* and *in vitro*. Compared to our previous data showing the effect of serial loss of SP sites from the C-terminal end of the protein *in vitro*, we now show, *in vivo*, that the reciprocal mutation series from the N-terminus of the protein behaves in a similar way. We can clearly now demonstrate that it is neither specific SP sites, nor their location in a particular protein domain, but rather it is the absolute number of phosphorylation sites, that determines Ngn2 protein activity.

### Ngnproteins are predicted to be intrinsically disordered outside the bHLH domain

The results presented above, taken together with data in [38] and [39], strongly support the hypothesis that the ability of xNgn2 to drive neuronal differentiation is regulated by modification on multiple SP sites and that it is the number of SP sites available for modification and not their position that is the prime determinant of neuronal differentiation activity. Structural consequences of phosphorylation could suggest a molecular mechanism for the multi-site phospho-regulation of Ngn2 activity but the structure of phosphorylated regions of Ngn2 has not been explored.

**Figure 3:**
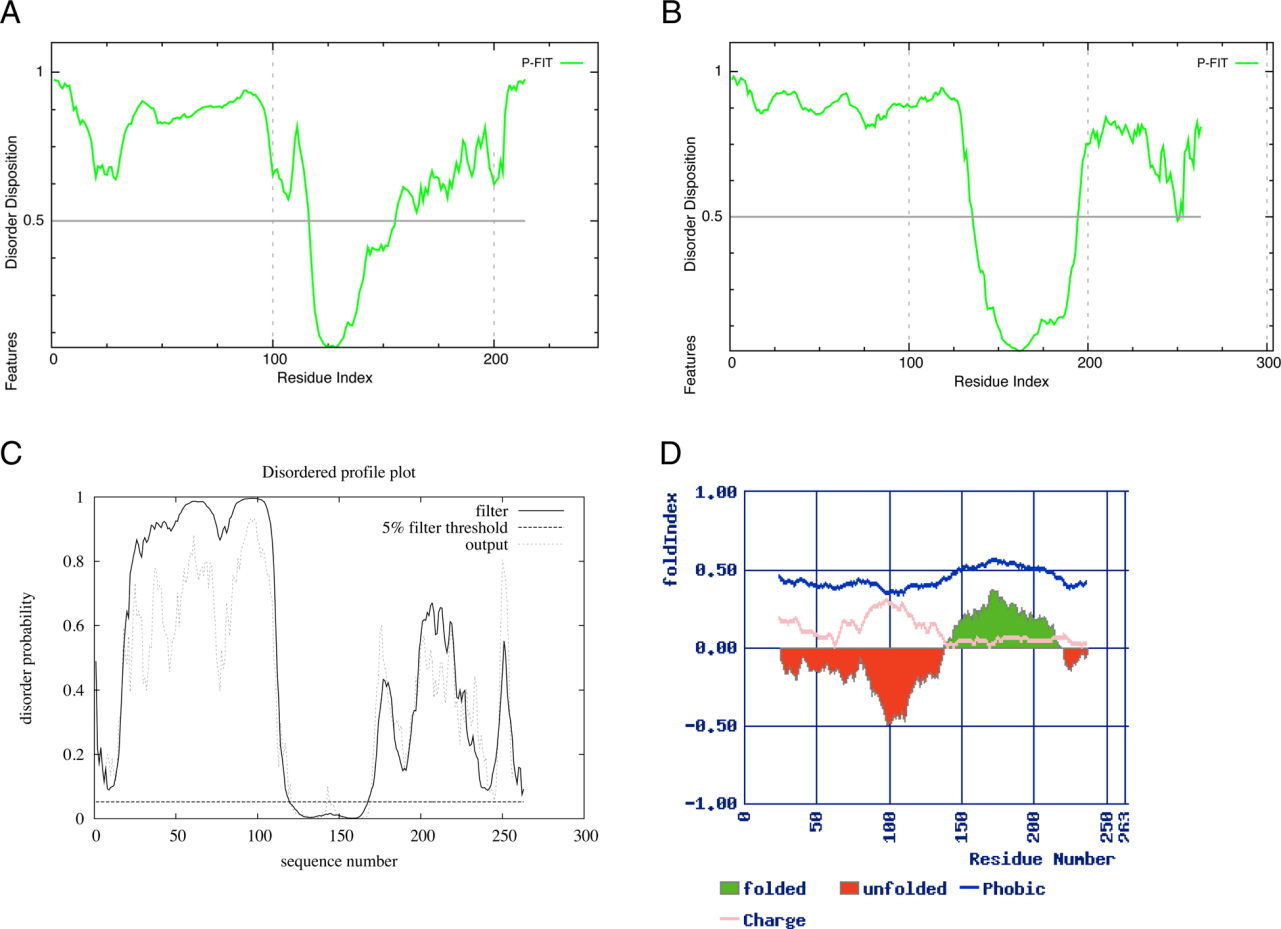
**The terminal regions of Ngn2 are predicted to be intrinsically disordered.** PONDR-FIT disorder predictions of **(A)** xNgn2 and **(B)** mNgn2. **(C)** DISOPRED2 and **(D)** FoldIndex disorder predictions of mNgn2.

By analogy with other bHLH proteins, it seems very likely that the N- and C-terminal regions of Ngn2 are intrinsically disordered. Ngn2 exhibits some of the features of ID proteins: there are a large number of charged residues leading to a low level of hydrophobicity [27]; and the protein runs at a higher molecular mass (around 36 kDa) than its calculated molecular mass (23.4 kDa) [18]. Computational analysis of the extent of protein disorder using PONDR-FIT (available from www.DisProt.org/pondr-fit.php[42]), predicts a high degree of disorder in the N- and C-terminal regions of *Xenopus* (Figure 3A) and mouse (Figure 3B) Ngn2, with a highly ordered section in the middle of the sequence corresponding to the bHLH domain. Both *Xenopus* and mouse Ngn2 exhibit low sequence conservation in the N- and C-terminal regions (see above and Additional file 1: Figure S1) but these regions in both proteins are predicted to be similarly highly disordered (Figure 3A, B), Further disorder predictions were carried out with mouse Ngn2 in order to compare the results from various predictors.

DISOPRED2 (http://bioinf.cs.ucl.ac.uk/disopred/, [43]) is a prediction algorithm that has been developed using empirically determined standards from experimental datasets. DISOPRED2 predicts that mNgn2 contains highly disordered N- and C-terminal regions flanking a folded region (Figure 3C), in agreement with the PONDR-FIT prediction (Figure 3B). FoldIndex (http://bip.weizmann.ac.il/fldbin/findex, [44]) uses the hydrophobicity and charge of the residues within the amino acid sequence to predict disordered regions and in agreement with the other algorithms, predicts disordered regions in mNgn2 at the N- and C-terminal regions interspaced by a folded region (Figure 3D). However, compared to the previous predictions (Figure 3B, C), FoldIndex predicts the folded region to be shifted towards the N-terminus and to be much larger. This correlates with the distribution of hydrophobic and charged residues in mNgn2 (Additional file 1: Figure S1). Therefore whilst Ngn2, and other bHLH proteins, may not be disordered across their entire length, empirical observations relating to the difficulties of protein purification [33], together with the disorder predictions for mNgn2 described above, suggest that intrinsically disordered regions are present.

NMR spectroscopy runs into a size problem for folded proteins because of increased tumbling time with concomitant enhanced line broadening of the signals [45]. By contrast, in ID proteins the fast molecular internal dynamics contribute to longer relaxation times compared to folded proteins of the same size, and result in narrow resonances that allow good detection and resolution [45]. Using this property of the NMR signal, we set out to determine whether we could detect the disordered regions of Ngn2 and phosphorylation events therein.

### Characterization of mouse Ngn2 conformation in solution and mapping of mouse Ngn2 phosphorylation sites using NMR spectroscopy

**Figure 4:**
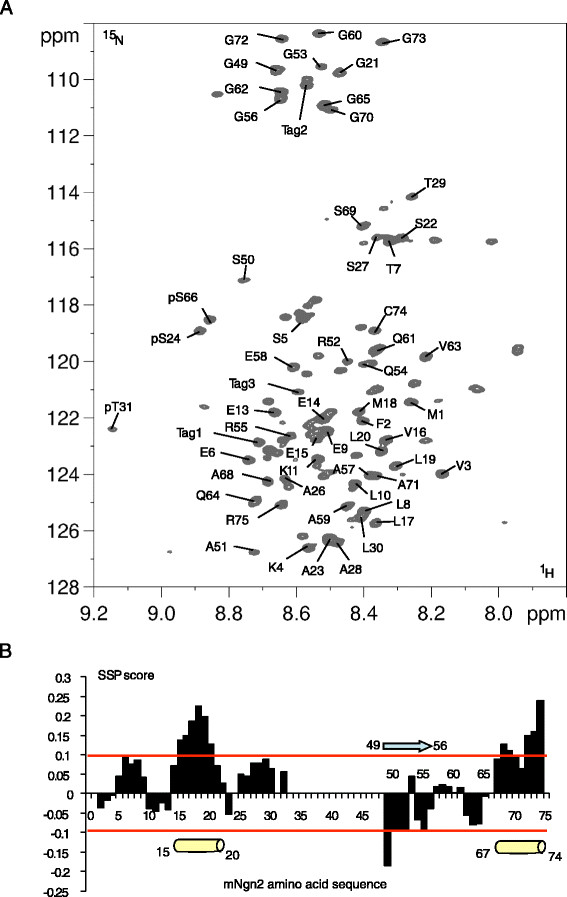
**Annotated**^**1**^**H,**^**15**^**N 2D spectra of phosphorylated**^**15**^**N-mNgn2 showing altered structural positions of phosphorylated residues. (A)** Detail of overlayed 2D [^1^H, ^15^N] HSQC spectra of ^15^N mNgn2 phosphorylated with cycA/CDK2. Resonances corresponding to phosphorylated Ser and Thr residues are labelled. **(B)** Secondary Structure Propensity (SSP, [46]) along the mNgn2 sequence calculated based on the available CA and CB chemical shifts (Additional file 4: Table S1). SSP scores correspond to a calculated percentage of occupancy: the 0.1/-0.1 thresholds, here represented as red lines, being empirically proposed for significance. Regions with positive values are associated with a preferential ?-helical conformation, here presented as cylinders. Regions with negative values are associated with a preferential extended conformation, here presented as arrows.

In order to directly confirm SP or TP sites in mNgn2 as targets for phosphorylation and to obtain further information on its secondary structure, a bacterially-expressed GST-mNgn2 fusion protein was ^15^N, or ^15^N, ^13^C isotopically-labelled for NMR spectroscopy analysis. Mouse Ngn2 was chosen, as although both mouse and *Xenopus* Ngn2 have been well-characterized [38],[39], previous attempts to purify *Xenopus* Ngn2 had been unsuccessful and it was hoped that mouse Ngn2, codon-optimized for bacterial expression, might prove more tractable (see Methods). Despite the limited solubility of full-length phosphorylated mNgn2 (see below), after cleavage of the GST carrier protein, we were still able to obtain a 2D ^1^H, ^15^N HSQC spectrum with close to 100 resonances (Figure 4, Additional file 3: 2D NMR dataset). The poor dispersion of the signal on the ^1^H scale indicates that the detected regions are disordered in nature, confirming the primary sequence-based predictions (Figure 3).

To investigate phosphorylation events in its disordered regions, recombinant mNgn2 protein was phosphorylated *in vitro* by incubation with recombinant CyclinA/CDK2 kinase, which was previously shown to phosphorylate xNgn2 ([38] and unpublished data) and regulates xNgn2 activity *in vivo*[38]. Analysis of the phosphorylated forms of mNgn2 using 2D NMR spectroscopy showed 3 new resonances that were absent in the 2D spectrum of the unphosphorylated form of mNgn2, in the region expected for pSer and pThr residues (Figure 4A, Additional file 3: 2D NMR dataset).

Assignment of the backbone atom resonances of mNgn2 was performed based on 3D experiments on the ^15^N, ^13^C doubly-labelled protein (Additional file 4: Table S1, Additional file 5: 3D NMR dataset). This assignment does not cover the full-length protein: the detected domains correspond to residues 1 to 31 and 49 to 75, containing two SP sites and one TP site. Comparison with the secondary structure prediction based on the primary sequence confirms that these regions of the protein are located outside of the bHLH domain and are not predicted to adopt stable secondary structures (Figure 3 and Additional file 1: Figure S1). Although these regions are disordered, closer examination of their chemical shifts by comparison with databases of chemical shifts of strictly random coil polymers [46] would suggest a local tendency to adopt transient secondary structure. Using the secondary structure propensity analysis, we observed a positive deviation of the experimental chemical shifts in segments aa15-20 and aa67-74 of the protein, consistent with the adoption of transient alpha-helical structure. However, a negative deviation in the C-terminal region between aa49-56 is indicative of a tendency to adopt an extended conformation (Figure 4B). The phosphorylated sites therefore reside in regions of high intrinsic disorder.

We next used the carbon signals in the 3D experiments to identify the phosphorylated residues [47],[48]. The CA and CB chemical shifts allow discrimination of a pS from a pT residue [49], while the CA-1 and CB-1 resonances will inform on the nature of the amino acid at the N-terminus (Additional file 4: Table S1). Cdks are proline-directed kinases and a proline at the i?+?1 position will alter the CA chemical shift by a +2 ppm deviation compared to the chemical shift expected for a pS or pT [50]. Phosphorylated residues were thus identified as pS24, pT31 and pS66, which are SP and TP sites conserved between xNgn2 and mNgn2 (Figure 4A and Additional file 1: Figure S1).

This is the first structural information obtained by NMR for full-length Ngn2 protein and it directly demonstrates the intrinsic disorder inherent to the protein, a characteristic of Ngn2 previously only predicted by using protein folding computational algorithms. In addition we have directly shown that protein phosphorylation *in vitro* can be mediated by cyclinA/CDK2 within the intrinsically disordered domain.

## Discussion

Studies are revealing the remarkable plasticity of fully differentiated somatic cells when exposed to an exogenous set of transcription factors. From the now well established transcription factor cocktails that promote reprogramming to an induced pluripotent cell state [1] through to reprogramming to induced neurons [51], the power of specific transcription factor networks to determine cell fate even in the absence of the normal physiological environment or developmental stage is well accepted. However, such transcription factor networks must regularly be broken and replaced during embryonic development, implying a tight regulation of the transcription factors themselves and mechanisms for restraining their activities. Although post-translational methods of regulation, such as phosphorylation and protein degradation, are beginning to be more widely investigated [4], mechanisms integrating different forms of post-translational regulation have been poorly studied. We have previously described regulation of the proneural bHLH transcription factor Ngn2 by both ubiquitin-mediated proteolysis and cell cycle-mediated phosphorylation at SP sites [28],[35]-[39]. In this study, we have explored possible links between these modes of regulation and investigated in greater depth the regulation and structural consequences of Ngn2 by phosphorylation at SP sites.

By comparing the protein primary sequence of xNgn2 to homologues in other species (Additional file 1: Figure S1), it is clear that conservation of Ngn2 sequence outside of the bHLH domain is poor. Strikingly, however, conservation of SP sites in the C-terminal region is very high, and more so than the conservation of SP sites in the N-terminal region. As Ngn2 is a highly unstable protein, we investigated whether phosphorylation of these sites might influence protein half-life. Despite conservation, mutation of SP sites in the C-terminal region of xNgn2 had no significant effect on protein stability, nor did mutation of N-terminal SP sites (Figure 1A), indicating that phosphorylation on these sites does not regulate the intrinsic stability of the protein. However, assays of stability in this *in vitro* system do not preclude the possibility that phosphorylation of Ngn2 could indirectly influence protein stability, for instance by affecting its ability to associate with binding partners not present in egg extract. Both N- and C-terminal SP sites regulate the overall neurogenic activity of xNgn2 *in vivo* (Figure 1B, C) but our data taken together indicate (as only suggested indirectly in previous work) that control of intrinsic protein stability is not the major mechanism by which phosphorylation at SP sites of xNgn2 acts to control neuronal differentiation activity.

We went on to expand upon our previous observations [38],[39] and to explore further whether the position of SP sites has a major influence on their ability to regulate the neuronal differentiation activity of xNgn2 by using phosphomutant Ngn2s *in vivo* in developing embryos. In contrast to mNgn2, where mutation of its two N-terminal SP sites did not significantly enhance its neurogenic activity *in vitro* in P19 cells, SP sites in both the N- and C-terminal regions of xNgn2 contribute to regulation of its neuronal differentiation activity *in vivo* in *Xenopus* embryos (Figure 1B, C and [38]). Our previous observation *in vitro* that it is the number and not position of SP sites which is the determinant of Ngn2 neuronal differentiation activity [38] was confirmed *in vivo* in *Xenopus* embryos using a series of xNgn2 mutants in which SP sites had been cumulatively changed to AP sites, beginning from the N-terminus (cumulative SP site mutant series, see Additional file 2: Figure S2 for more information, Figure 2). These results, using assays of activity *in vivo*, clearly reinforce a model suggested by *in vitro* data [38] that it is the number, and not position, of phosphorylation events at SP sites in Ngn2 that regulate its neuronal differentiation activity.

As it is not the precise location but the number of phospho-sites available that regulates Ngn2 activity, this led us to consider whether structural plasticity associated with intrinsic disorder might facilitate such regulation. In this regard, previous studies suggested that these regions might be intrinsically disordered [18],[27],[33]. ID proteins show an increased rate of evolution in disordered regions and both *Xenopus* and mouse Ngn2 exhibit low sequence conservation in the N- and C-terminal regions (Additional file 1: Figure S1).

The purification of bHLH proteins is problematic as illustrated by the work of Aguado-Lllera *et al*. [33]. The authors attempted to purify the bHLH domain of Ngn1 using His-tagged, GST-tagged, maltose binding protein-tagged, biotin-tagged and thioredoxin-tagged protein constructs in BL21, Rosetta, C41 and BL21pLys cells with induction at several different temperatures and with varying concentrations of IPTG. Only one combination appeared to work at all but the protein was found to be partially degraded. The authors finally turned to chemical synthesis of the bHLH domain of Ngn1 instead. Indeed, bHLH protein crystal structure determination is usually performed on a protein devoid of the N- and C-terminal regions (i.e., regions outside of the bHLH domain [31]) as these regions have previously been predicted to be disordered. However, some evidence exists that even within the bHLH domain bound to both its heterodimeric binding partner and E-box DNA there is still a tendency towards disorder [33]. Disorder-predicting programs are based upon protein sequence information alone and the *in vivo* environment is not yet accurately reproducible *in silico*. Nevertheless, the predictions performed here (Figure 3) do correlate well for both *Xenopus* and mouse Ngn2 and suggest that a high degree of disorder is likely, particularly in the N- and C-terminal regions. The FoldIndex prediction (Figure 3D) correlates least with the other predictions. However, FoldIndex places a greater bias upon the hydrophobicity and charge of the residues in the peptide sequence [44] and whilst this is usually a good predictor of intrinsic disorder [27] there may be circumstances under which it is insufficient.

To directly investigate the level of disorder within the regions of Ngn2, we attempted to purify mNgn2 and investigate its tertiary structure and phosphorylation by NMR. Unsurprisingly, the purification of mNgn2 proved to be difficult; previous attempts to purify xNgn2, using both bacterial expression and baculovirus systems had been largely unsuccessful (data not shown). Sarkosyl-based treatment [52] resulted in the liberation of some His-mNgn2 from the insoluble fraction of the cell lysate. Solubility of the protein was greatly improved by using a GST-tag and we were able to isolate GST-mNgn2 from the soluble fraction instead (data not shown). Despite these difficulties, we were able to purify a sufficient quantity of protein to obtain unambiguous NMR spectra, although signals originate from only the N-terminal region of the protein. The NMR spectra for mNgn2 demonstrated that the N-terminal region of the protein was disordered, and that serines 24 and 66 and threonine 31 are phosphorylated *in vitro* by cyclinA/CDK2 (Figure 4A). That the NMR results comprise spectra allowing the identification of phosphorylated SP and TP sites in the N-terminal region, conserved between xNgn2 and mNgn2, illustrates not only direct phosphorylation of SP sites by cyclinA/CDK2 *in vitro* but also that this occurs in the disordered N-terminal region. This structural evidence for the disordered N-terminal domain, and the phosphorylation which modifies it, provide a direct demonstration of phosphorylation of Ngn2, and compliment previous work inferring phosphorylation by using extract systems *in vitro* and by using activity assays of phospho-mutant Ngn2 *in vivo*.

Our data demonstrate that the N-terminal region is intrinsically disordered and argue for a role for ID regions as integrators of signaling leading to tight regulation of the neuronal differentiation activity of Ngn2. Intrinsically disordered regions may well be enriched in post-translational modifications [26], making them ideal for integration of different signaling events and post-translational mechanisms of regulation. Such regulation is critical for normal development and the role of disordered regions highlights an alternative to the more widely established mechanism of switching between rigidly structured forms upon post-translational modification. Our data support our previous findings that it is the number, and not position, of phosphorylation sites available that controls Ngn2 neuronal differentiation activity [38],[39]. This is similar to another process involving cell cycle regulation in the process of differentiation, namely the role of the Ste5 protein in MAP Kinase signaling in yeast [53].

## Conclusions

Phosphorylation at serine-proline (SP) sites in Ngn2 occurs in both the N- and C-terminal regions, located either side of the bHLH domain, and this phosphorylation does not alter the intrinsic stability of Ngn2 protein. Multi-site phosphorylation of SP sites inhibits the neuronal differentiation activity of Ngn2 protein, but it is the total number of phospho-sites available, rather than their precise sequence location or approximate location within a particular domain within the Ngn2 protein, that controls the ability of Ngn2 to drive neuronal differentiation *in vivo* in *Xenopus* embryos. We find that the N-and C-terminal regions of Ngn2 are intrinsically disordered and we have taken advantage of the disordered nature of the mNgn2 protein, and the ability to gain structural information about the N-terminal domain in particular, to observe phosphorylation by cyclinA/CDK2 kinase *in vitro* on an intrinsically disordered protein using protein NMR.

## Methods

### Animal care

*Xenopus laevis* were housed, bred, and treated according to the guidelines approved by the UK home office under the animal (Scientific Procedures) Act 1986. All animal work has been carried out under UK Home Office Licence and has passed an Institutional ethical review committee assessment, undertaken by the Animal Welfare and Ethical Review Committee (AWERC) at the University of Cambridge.

### Cloning

Point-mutant constructs were made by site-directed mutagenesis (Stratagene) and cloned into pCS2+ as described previously [28],[35]. Subcloning was carried out using standard methods.

### GST-tagged Ngn2

A DNA construct of mNgn2, codon optimised for bacterial expression, was produced by Genecust (Luxembourg) in a pUC vector. This was placed into the pGEX (N-terminal GST-tagging [54]) vector by subcloning by restriction digest and ligation [55].

### *In vitro* translation

*In vitro* translation was carried out using the TNT® SP6 quick coupled transcription/translation system (Promega), in the presence of ^35^S-methionine (GE Healthcare), according to the manufacturer¿s instructions.

### *In vitro* transcription of RNA

RNA for microinjection (mRNA) or as an antisense probe for *in situ* hybridization was transcribed *in vitro* using constructs with a linearised pCS2+ vector template. Transcription was performed as described previously [38] using either the SP6 mMessage mMachine kit (Ambion) for mRNA or T3 polymerase (Roche) for antisense probe. Digoxigenin (DIG) labelling of the antisense probe was performed as described previously [38].

### *Xenopus* extracts

Activated mitotic egg extract was prepared as described previously [35].

### Degradation assays

Degradation assays were carried out as described previously [28],[35],[37].

### *In situ* hybridization

*In situ* hybridization was carried out as described previously using neural ?-tubulin antisense RNA as a probe [38],[39].

### Quantitative PCR

Quantitative real-time PCR (qPCR) was performed as described previously [38]. Briefly, *Xenopus* embryos were injected with 20 pg of mRNA at the 1 cell-stage and embryos were allowed to develop to Nieuwkoop and Faber stage 15 [56]. Samples were pooled such that 5 embryos for each condition underwent mRNA extraction with the RNeasy Mini kit (Qiagen) according to manufacturer¿s instructions. Reverse transcription to form cDNA was performed with oligodT primers and the Transcriptor High Fidelity cDNA Synthesis kit (Roche) according to manufacturer¿s instructions.

### Clustal Wsequence alignment

Clustal W2 analysis was carried out to align protein sequences [57].

### Protein disorder prediction

Protein sequences were submitted to PONDR-FIT (available from www.DisProt.org/pondr-fit.php, [42]), DISOPRED2 (http://bioinf.cs.ucl.ac.uk/disopred/, [43]) and FoldIndex (http://bip.weizmann.ac.il/fldbin/findex, [44]) for disorder prediction.

### Secondary structure prediction

Secondary Structure Propensity (SSP, [46]) along the mNgn2 sequence was calculated based on the available CA and CB chemical shifts from NMR data.

### Protein purification

GST-mNgn2 was expressed in BL21 (DE3) cells transformed with ampicillin-resistant plasmids and grown in M9-based semi-rich medium (M9 medium (50 mM Na_2_HPO_4_, 15 mM KH_2_PO_4_, 8.5 mM NaCl) supplemented with MEM, 1 mM MgSO_4_, 100 ?M CaCl_2_, 1 g l^?1^^15^?N-NH_4_Cl, 2 g l^?1^^13^C_6_-D-glucose (when ^13^C labelling required, otherwise 4 g l^?1^ unlabelled glucose (Sigma) used), 0.7 g l^?1^ Isogro ^13^C, ^15^N powder growth medium (Sigma), 100 ?g ml^?1^ ampicillin) at 37°C to an OD_600_ of 0.6. Protein expression was induced with 0.4 mM IPTG at 20°C overnight. Harvested cells were lysed using lysozyme and sonication. Proteins were purified on a glutathione-bead containing column (Amersham) using an AKTA FPLC purifier (GE Healthcare) and eluted by cleavage of the GST tag from the protein using PreScission Protease (GE Healthcare) overnight at 4°C and elution in 1 × PBS supplemented with 2 mM EDTA.

### In vitro phosphorylation of mNgn2

mNgn2 was phosphorylated using recombinant CyclinA3/Cdk2 [58] protein in 5 mM ATP, 12.5 mM MgCl_2_, 50 mM HEPES pH 8.0, 55 mM NaCl, 5 mM DTT, at 30°C for 5 hours [59] before passing through a G25 desalting resin in a Zeba spin column (Pierce) to buffer exchange into NMR buffer (50 mM Tris, 25 mM NaCl, 2.5 mM EDTA and 2 mM DTT, pH 6.8).

### NMR

1 mM D_4_-TMSP (TriMethyl Silyl Propionate), used as a proton chemical shift internal reference (0 ppm or part per million), and 5% D_2_O were added to protein samples. [^1^H, ^15^N] HSQC 2D spectra were recorded at 277 K on a Bruker 600 spectrometer equipped with a triple resonance cryogenic probehead (Bruker, Karlsruhe, Germany). Assignment was performed on a sample of doubly labelled protein at 150 ?M, using classical pairs of 3D experiments. Spectra were processed using Bruker TOPSPIN 2.1 (Bruker, Karlsruhe, Germany). Peak picking was performed using Sparky (T. D. Goddard and D. G. Kneller, SPARKY 3, University of California, San Francisco).

## Availability of supporting data

The data sets supporting the results of this article are included within the article and in its additional files. NMR datasets for 2D and 3D experiments are labelled as ¿2DNMR¿ and ¿3DNMR¿ respectively.

## Abbreviations

Ngn2: Neurogenin2

x: *Xenopus laevis*

m: *Mus musculus*

h: *Homo sapiens*

bHLH: Basic Helix-Loop-Helix

ID: Intrinsic disorder

IVT: *in vitro* translation

pI: Isoelectric point

## Competing interests

The authors declare that they have no competing interests.

## Authors¿ contributions

GM carried out degradation assays and bioinformatic analysis. CH carried out *in situ* hybridizations and qPCR analysis. GM and IL purified protein and carried out the NMR studies, and IL analysed the data. AP and GL conceived the study, and participated in its design and coordination. GM, CH, IL and AP wrote the manuscript. All authors have read and approved the final manuscript.

## Additional files

## Supplementary Material

Additional file 1: Figure S1. SP sites are conserved in the C-terminal domain. Ngn2 protein sequences from the NCBI database were aligned using ClustalW2. The bHLH domain is indicated by the green box and SP sites conserved in over half of all species are indicated by the red boxes. Click here for file

Additional file 2: Figure S2. SP site xNgn2 mutants schematic. Illustration of the various SP site mutants of xNgn2 used in this study. Click here for file

Additional file 3: **2D NMR dataset.** Raw data for 2D NMR experiments. Click here for file

Additional file 4: Table S1. mNgn2 CA and CB chemical shifts. Available CA and CB chemical shifts for visible residues. Click here for file

Additional file 5: **3D NMR dataset.** Raw data for 3D NMR experiments. Click here for file
