# Supplementary material for: Phosphorylation in intrinsically disordered regions regulates the activity of Neurogenin2
Source: BMC Biochem. 2014 Nov 6;15:24. doi: 10.1186/s12858-014-0024-3 (PMC4422318; doi:10.1186/s12858-014-0024-3)

Supplementary Figure 1

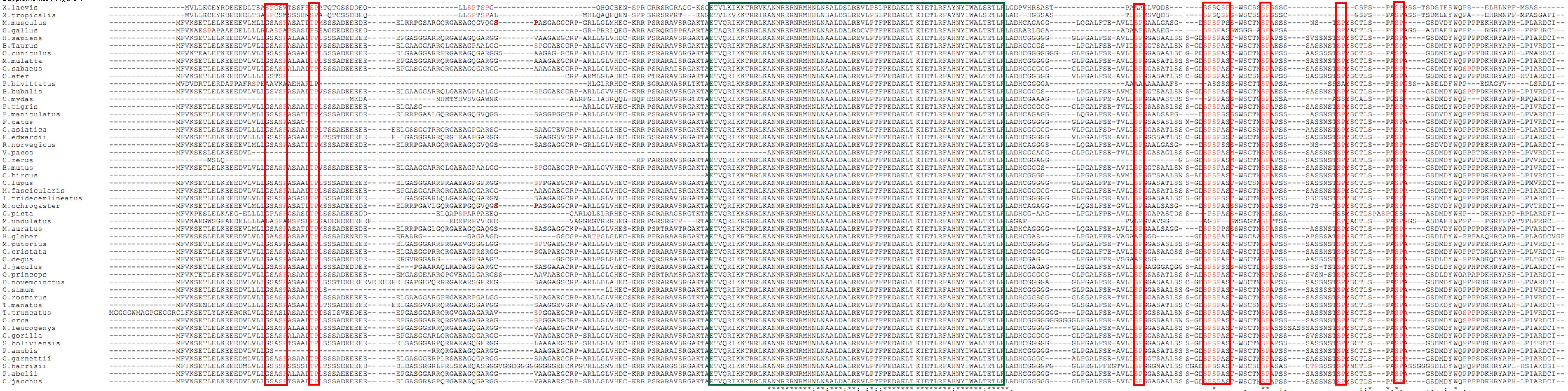

Supplement: Additional file 1: Figure S1. — SP sites are conserved in the C-terminal domain. Ngn2 protein sequences from the NCBI database were aligned using ClustalW2. The bHLH domain is indicated by the green box and SP sites conserved in over half of all species are indicated by the red boxes. [file s12858-014-0024-3-S1.pdf]
