# Supplementary figures and images for: Phosphorylation in intrinsically disordered regions regulates the activity of Neurogenin2
Source: BMC Biochem. 2014 Nov 6;15:24. doi: 10.1186/s12858-014-0024-3 (PMC4422318; doi:10.1186/s12858-014-0024-3)

SUPPLEMENTARY FIGURE 2

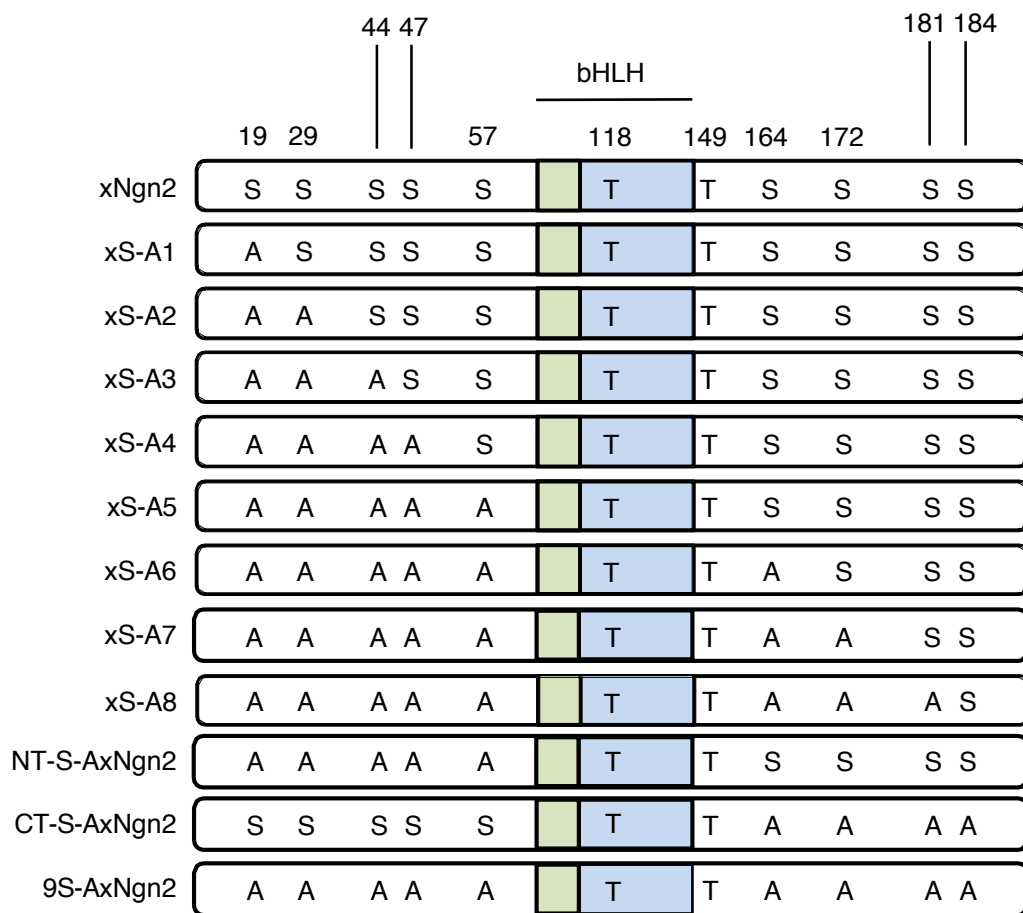

Supplement: Additional file 2: Figure S2. — SP site xNgn2 mutants schematic. Illustration of the various SP site mutants of xNgn2 used in this study. [file s12858-014-0024-3-S2.pdf]
