# Supplementary material for: Phosphorylation in intrinsically disordered regions regulates the activity of Neurogenin2
Source: BMC Biochem. 2014 Nov 6;15:24. doi: 10.1186/s12858-014-0024-3 (PMC4422318; doi:10.1186/s12858-014-0024-3)
Supplement: Additional file 4: Table S1. — mNgn2 CA and CB chemical shifts. Available CA and CB chemical shifts for visible residues. [file s12858-014-0024-3-S4.doc]

**Additional file 1: Table S1**

| AA Residue | HN (ppm) | N (ppm) | CA (ppm) | CB (ppm) |
| --- | --- | --- | --- | --- |
| **M1** | 8.26 | 121.4 | 55.4 | 32.7 |
| **F2** | 8.41 | 122.1 | 58.0 | 39.6 |
| **V3** | 8.17 | 124 | 62.1 | 33.1 |
| **K4** | 8.56 | 126.6 | 57.0 | 33.0 |
| **S5** | 8.58 | 118.5 | 58.6 | 64.0 |
| **E6** | 8.74 | 123.5 | 56.9 | 30.2 |
| **T7** | 8.32 | 115.7 | 62.4 | 69.9 |
| **L8** | 8.40 | 125.3 | 55.3 | 42.3 |
| **E9** | 8.51 | 122.5 | 56.6 | 30.3 |
| **L10** | 8.43 | 124.4 | 55.2 | 42.3 |
| **K11** | 8.53 | 123.5 | 56.3 | 33.2 |
| **E12** | 8.68 | 123.2 | 56.3 | 30.3 |
| **E13** | 8.66 | 121.8 | 57.1 | 30.1 |
| **E14** | 8.51 | 122 | 56.9 | 30.4 |
| **E15** | 8.54 | 122.8 | 57.1 | 30.4 |
| **V16** | 8.33 | 122.8 | 63.2 | 32.5 |
| **L17** | 8.36 | 125.3 | 55.6 | 42.2 |
| **M18** | 8.41 | 121.8 | 55.5 | 32.6 |
| **L19** | 8.30 | 123.7 | 55.3 | 42.2 |
| **L20** | 8.35 | 123.2 | 55.6 | 42.2 |
| **G21** | 8.47 | 109.8 | 45.3 | - |
| **S22** | 8.27 | 115.7 | 58.2 | 64.1 |
| **A23** | 8.50 | 126.4 | 52.8 | 19.3 |
| **pS24** | 8.89 | 119.9 | 56.3 | 64.8 |
| **P25** |  |  | 63.8 | 32.0 |
| **A26** | 8.63 | 124.2 | 52.9 | 19.0 |
| **S27** | 8.36 | 115.6 | 58.7 | 64.0 |
| **A28** | 8.48 | 126.3 | 52.8 | 19.3 |
| **T29** | 8.26 | 114.2 | 62.2 | 69.8 |
| **L30** | 8.40 | 125.4 | 55.3 | 42.4 |
| **pT31** | 9.14 | 123 | 61.0 | 11.2 |
| **P48** |  |  | 63.6 | 32.1 |
| **G49** | 8.66 | 109.7 | 45.3 | - |
| **S50** | 8.76 | 117.1 | 57.8 | 66.1 |
| **A51** | 8.73 | 126.8 | 53.2 | 18.8 |
| **R52** | 8.45 | 120 | 56.7 | 30.7 |
| **G53** | 8.52 | 109.6 | 45.5 | - |
| **Q54** | 8.39 | 120.1 | 55.9 | 29.5 |
| **R55** | 8.63 | 122.6 | 56.4 | 30.8 |
| **G56** | 8.65 | 110.7 | 45.3 | - |
| **A57** | 8.38 | 124.1 | 52.9 | 19.1 |
| **E58** | 8.61 | 120.2 | 56.8 | 29.9 |
| **A59** | 8.45 | 125.2 | 53.1 | 19.1 |
| **G60** | 8.53 | 108.4 | 45.4 | - |
| **Q61** | 8.37 | 119.6 | 56.0 | 29.4 |
| **G62** | 8.64 | 110.4 | 45.3 | - |
| **V63** | 8.22 | 119.9 | 62.7 | 32.7 |
| **Q64** | 8.73 | 125 | 55.8 | 29.6 |
| **G65** | 8.52 | 110.9 | 44.8 | - |
| **pS66** | 8.86 | 118.5 | 56.2 | 65.0 |
| **P67** |  |  | 63.8 | 32.0 |
| **A68** | 8.68 | 124.5 | 52.9 | 18.9 |
| **S69** | 8.40 | 115.2 | 58.9 | 63.9 |
| **G70** | 8.50 | 111.1 | 45.5 | - |
| **A71** | 8.36 | 124.1 | 53.1 | 19.1 |
| **G72** | 8.64 | 108.6 | 45.6 | - |
| **G73** | 8.35 | 108.7 | 45.3 | - |
| **C74** | 8.37 | 118.9 | 58.6 | 28.0 |
| **R75** | 8.65 | 125.1 | 54.2 | 30.1 |
